# 2-[(4-Chloro­anilino)meth­yl]phenol

**DOI:** 10.1107/S1600536811029047

**Published:** 2011-07-23

**Authors:** Li-Zhuang Chen

**Affiliations:** a School of Biology and Chemical Engineering, Jiangsu University of Science and Technology, Zhenjiang 212003, People’s Republic of China

## Abstract

In the title compound, C_13_H_12_ClNO, the dihedral angle between the two benzene ring planes is 68.71 (8)°. In the crystal, mol­ecules are linked by pairs of O—H⋯N hydrogen bonds into inversion dimers, which are further linked by intermolecular N—H⋯O interactions into a chain running parallel to the *a* axis.

## Related literature

For the synthesis of the title compound, see: Noda (1959 ▶). For related structures, see: Liu *et al.* (2007 ▶); Qu *et al.* (2007 ▶).
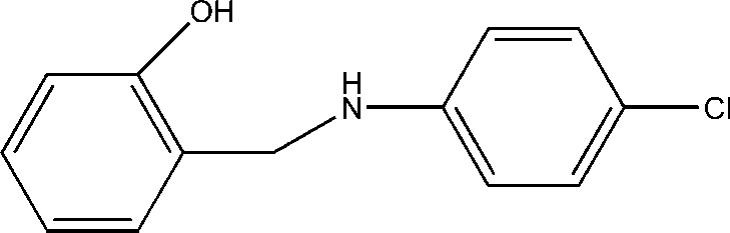


## Experimental

### 

#### Crystal data

C_13_H_12_ClNO
*M* *_r_* = 233.69
Triclinic, 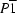

*a* = 5.5842 (11) Å
*b* = 7.9485 (16) Å
*c* = 13.023 (3) Å
α = 86.87 (3)°
β = 89.12 (3)°
γ = 88.65 (3)°
*V* = 577.0 (2) Å^3^
*Z* = 2
Mo *K*α radiation
μ = 0.31 mm^−1^
*T* = 298 K
0.40 × 0.30 × 0.20 mm

#### Data collection

Rigaku SCXmini diffractometer
Absorption correction: multi-scan (*CrystalClear*; Rigaku, 2005 ▶) *T*_min_ = 0.895, *T*_max_ = 0.940
5968 measured reflections
2637 independent reflections
1383 reflections with *I* > 2σ(*I*)
*R*_int_ = 0.061

#### Refinement

*R*[*F* ^2^ > 2σ(*F* ^2^)] = 0.068
*wR*(*F* ^2^) = 0.186
*S* = 0.98
2637 reflections
153 parameters
2 restraints
H atoms treated by a mixture of independent and constrained refinement
Δρ_max_ = 0.22 e Å^−3^
Δρ_min_ = −0.26 e Å^−3^

### 

Data collection: *CrystalClear* (Rigaku, 2005 ▶); cell refinement: *CrystalClear*; data reduction: *CrystalClear*; program(s) used to solve structure: *SHELXS97* (Sheldrick, 2008 ▶); program(s) used to refine structure: *SHELXL97* (Sheldrick, 2008 ▶); molecular graphics: *SHELXTL* (Sheldrick, 2008 ▶); software used to prepare material for publication: *SHELXL97*.

## Supplementary Material

Crystal structure: contains datablock(s) I, global. DOI: 10.1107/S1600536811029047/pv2430sup1.cif

Structure factors: contains datablock(s) I. DOI: 10.1107/S1600536811029047/pv2430Isup2.hkl

Supplementary material file. DOI: 10.1107/S1600536811029047/pv2430Isup3.cml

Additional supplementary materials:  crystallographic information; 3D view; checkCIF report

## Figures and Tables

**Table 1:** Hydrogen-bond geometry (Å, °)

*D*—H⋯*A*	*D*—H	H⋯*A*	*D*⋯*A*	*D*—H⋯*A*
N1—H1*B*⋯O1^i^	0.86 (1)	2.20 (1)	3.038 (3)	165 (3)
O1—H1*A*⋯N1^ii^	0.85 (1)	1.93 (1)	2.777 (3)	171 (3)

Symmetry codes: (i) 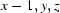; (ii) 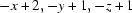.

**Fig. 1.**
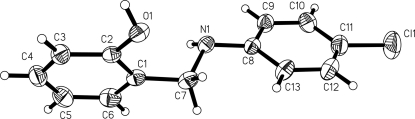
A view of the title compound with atomic labels. Displacement ellipsoids were drawn at the 30% probability level.

**Fig. 2.**
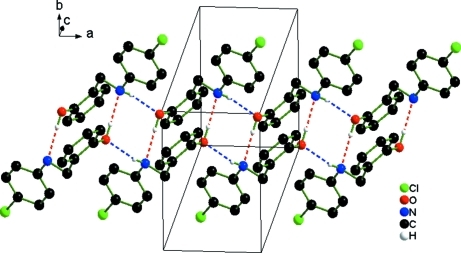
The unit cell packing of the title compound viewed along the a-axis. Hydrogen bonds are drawn as dashed lines and the H-atoms not involved in hydrogen bonding have been excluded for clarity.

## supplementary crystallographic information

### Comment

In the title compound (Fig. 1), which was synthesized by the reduction of 2-((4-chlorophenylimino)methyl)phenol, the dihedral angle between the two benzene ring planes is 68.71 (8)°. In the crystal structure, the molecules are linked by intermolecular N—H···O, and O—H···N hydrogen bonds into a one-dimensional chain along the *a*-axis (Tab. 1 & Fig. 2).

The crystal structure of compounds closely related to the title molecule, eg., 2-[(4-chlorophenyl)aminomethyl]-6-methoxyphenol (Liu *et al.*, 2007) and 2-(anilinomethyl)phenol (Qu *et al.*, 2007) have been reported.

### Experimental

The title compound was synthesized by the reaction of 2-((4-chlorophenylimino)- methyl)phenol (2.31 g, 10 mmol) with NaBH_4_ (0.38 g, 10 mmol) in 50 ml methanol according to the reported method (Noda, 1959). Crystals were obtained from an ethanolic (95%) solution by slow evaporation at room temperature.

### Refinement

H atoms bonded to C were placed at calculated positions and were included in the refinement in the riding-model approximation, with C—H = 0.93 and 0.97 Å, for aryl and methylene H atoms, respectively, and *U*_iso_(H) = 1.2*U*_eq_(C). The H atoms on N and O atoms were located form a difference map and were allowed to refine freely.

### Crystal data

**Table:**

C_13_H_12_ClNO	*Z* = 2
*M**_r_* = 233.69	*F*(000) = 244
Triclinic, *P*1	*D*_x_ = 1.345 Mg m^−^^3^
Hall symbol: -P 1	Mo *K*α radiation, λ = 0.71073 Å
*a* = 5.5842 (11) Å	Cell parameters from 4578 reflections
*b* = 7.9485 (16) Å	θ = 3.1–27.5°
*c* = 13.023 (3) Å	µ = 0.31 mm^−^^1^
α = 86.87 (3)°	*T* = 298 K
β = 89.12 (3)°	Prism, colorless
γ = 88.65 (3)°	0.40 × 0.30 × 0.20 mm
*V* = 577.0 (2) Å^3^	

### Data collection

**Table:**

Rigaku SCXmini diffractometer	2637 independent reflections
Radiation source: fine-focus sealed tube	1383 reflections with *I* > 2σ(*I*)
graphite	*R*_int_ = 0.061
Detector resolution: 13.6612 pixels mm^-1^	θ_max_ = 27.5°, θ_min_ = 3.1°
CCD_Profile_fitting scans	*h* = −7→7
Absorption correction: multi-scan (*CrystalClear*; Rigaku, 2005)	*k* = −10→10
*T*_min_ = 0.895, *T*_max_ = 0.940	*l* = −16→16
5968 measured reflections	

### Refinement

**Table:**

Refinement on *F*^2^	Primary atom site location: structure-invariant direct methods
Least-squares matrix: full	Secondary atom site location: difference Fourier map
*R*[*F*^2^ > 2σ(*F*^2^)] = 0.068	Hydrogen site location: inferred from neighbouring sites
*wR*(*F*^2^) = 0.186	H atoms treated by a mixture of independent and constrained refinement
*S* = 0.98	*w* = 1/[σ^2^(*F*_o_^2^) + (0.0821*P*)^2^] where *P* = (*F*_o_^2^ + 2*F*_c_^2^)/3
2637 reflections	(Δ/σ)_max_ = 0.002
153 parameters	Δρ_max_ = 0.22 e Å^−^^3^
2 restraints	Δρ_min_ = −0.26 e Å^−^^3^

### Special details

**Table:**

Geometry. All e.s.d.'s (except the e.s.d. in the dihedral angle between two l.s. planes) are estimated using the full covariance matrix. The cell e.s.d.'s are taken into account individually in the estimation of e.s.d.'s in distances, angles and torsion angles; correlations between e.s.d.'s in cell parameters are only used when they are defined by crystal symmetry. An approximate (isotropic) treatment of cell e.s.d.'s is used for estimating e.s.d.'s involving l.s. planes.
Refinement. Refinement of *F*^2^ against ALL reflections. The weighted *R*-factor *wR* and goodness of fit *S* are based on *F*^2^, conventional *R*-factors *R* are based on *F*, with *F* set to zero for negative *F*^2^. The threshold expression of *F*^2^ > σ(*F*^2^) is used only for calculating *R*-factors(gt) *etc*. and is not relevant to the choice of reflections for refinement. *R*-factors based on *F*^2^ are statistically about twice as large as those based on *F*, and *R*- factors based on ALL data will be even larger.

### Fractional atomic coordinates and isotropic or equivalent isotropic displacement parameters (Å^2^)

**Table:**

	*x*	*y*	*z*	*U*_iso_*/*U*_eq_	
Cl1	0.3943 (2)	0.26252 (15)	−0.02082 (7)	0.1029 (5)	
O1	1.2068 (3)	0.3737 (2)	0.53931 (14)	0.0477 (5)	
N1	0.6596 (4)	0.3048 (3)	0.41482 (17)	0.0427 (6)	
C2	1.0518 (4)	0.2978 (3)	0.6089 (2)	0.0395 (6)	
C1	0.8691 (4)	0.2033 (3)	0.5715 (2)	0.0431 (7)	
C8	0.5997 (4)	0.2890 (3)	0.3104 (2)	0.0420 (6)	
C3	1.0818 (5)	0.3070 (4)	0.7143 (2)	0.0504 (7)	
H3	1.2079	0.3668	0.7391	0.061*	
C7	0.8410 (5)	0.1848 (3)	0.4587 (2)	0.0500 (7)	
H7A	0.9936	0.2038	0.4239	0.060*	
H7B	0.7942	0.0707	0.4472	0.060*	
C6	0.7158 (5)	0.1240 (4)	0.6421 (3)	0.0593 (8)	
H6	0.5924	0.0604	0.6185	0.071*	
C9	0.3944 (5)	0.3687 (4)	0.2737 (2)	0.0519 (7)	
H9	0.2965	0.4278	0.3183	0.062*	
C13	0.7429 (5)	0.2015 (4)	0.2433 (2)	0.0545 (8)	
H13	0.8824	0.1469	0.2666	0.065*	
C11	0.4753 (6)	0.2735 (4)	0.1065 (2)	0.0611 (8)	
C10	0.3309 (6)	0.3627 (4)	0.1723 (2)	0.0621 (8)	
H10	0.1924	0.4181	0.1485	0.074*	
C4	0.9239 (5)	0.2272 (4)	0.7814 (2)	0.0589 (8)	
H4	0.9426	0.2351	0.8519	0.071*	
C12	0.6794 (6)	0.1947 (4)	0.1411 (2)	0.0642 (9)	
H12	0.7768	0.1361	0.0960	0.077*	
C5	0.7407 (6)	0.1367 (4)	0.7467 (2)	0.0633 (9)	
H5	0.6338	0.0842	0.7928	0.076*	
H1B	0.532 (3)	0.304 (4)	0.4527 (18)	0.058 (9)*	
H1A	1.248 (6)	0.469 (2)	0.560 (2)	0.087 (12)*	

### Atomic displacement parameters (Å^2^)

**Table:**

	*U*^11^	*U*^22^	*U*^33^	*U*^12^	*U*^13^	*U*^23^
Cl1	0.1313 (10)	0.1297 (10)	0.0496 (6)	−0.0068 (7)	−0.0160 (6)	−0.0149 (6)
O1	0.0482 (11)	0.0441 (12)	0.0517 (12)	−0.0070 (9)	0.0071 (9)	−0.0107 (10)
N1	0.0390 (13)	0.0442 (13)	0.0453 (14)	0.0033 (10)	0.0031 (10)	−0.0089 (11)
C2	0.0350 (14)	0.0364 (14)	0.0471 (16)	0.0057 (11)	0.0012 (12)	−0.0044 (12)
C1	0.0388 (14)	0.0375 (15)	0.0533 (17)	0.0010 (12)	−0.0030 (12)	−0.0051 (13)
C8	0.0393 (14)	0.0427 (15)	0.0446 (16)	−0.0074 (12)	0.0031 (12)	−0.0067 (12)
C3	0.0465 (16)	0.0530 (18)	0.0521 (18)	0.0009 (13)	−0.0077 (13)	−0.0044 (14)
C7	0.0440 (15)	0.0448 (16)	0.0622 (19)	0.0038 (13)	−0.0050 (13)	−0.0137 (14)
C6	0.0494 (18)	0.0526 (18)	0.076 (2)	−0.0090 (14)	−0.0072 (16)	−0.0022 (16)
C9	0.0522 (17)	0.0562 (18)	0.0474 (17)	0.0053 (14)	0.0028 (13)	−0.0061 (14)
C13	0.0460 (16)	0.0617 (18)	0.0578 (19)	0.0016 (14)	0.0018 (14)	−0.0214 (15)
C11	0.069 (2)	0.068 (2)	0.0471 (18)	−0.0102 (18)	−0.0011 (15)	−0.0069 (16)
C10	0.0618 (19)	0.069 (2)	0.0547 (19)	0.0018 (16)	−0.0088 (15)	0.0042 (16)
C4	0.067 (2)	0.064 (2)	0.0448 (18)	0.0051 (17)	−0.0008 (15)	0.0032 (15)
C12	0.068 (2)	0.070 (2)	0.057 (2)	−0.0049 (17)	0.0073 (16)	−0.0234 (17)
C5	0.063 (2)	0.066 (2)	0.060 (2)	−0.0059 (17)	0.0085 (16)	0.0116 (17)

### Geometric parameters (Å, °)

**Table:**

Cl1—C11	1.733 (3)	C7—H7B	0.9700
O1—C2	1.370 (3)	C6—C5	1.381 (4)
O1—H1A	0.853 (10)	C6—H6	0.9300
N1—C8	1.418 (3)	C9—C10	1.376 (4)
N1—C7	1.475 (3)	C9—H9	0.9300
N1—H1B	0.858 (10)	C13—C12	1.387 (4)
C2—C1	1.391 (4)	C13—H13	0.9300
C2—C3	1.391 (3)	C11—C12	1.358 (4)
C1—C6	1.383 (4)	C11—C10	1.381 (4)
C1—C7	1.495 (4)	C10—H10	0.9300
C8—C9	1.376 (4)	C4—C5	1.361 (4)
C8—C13	1.381 (4)	C4—H4	0.9300
C3—C4	1.374 (4)	C12—H12	0.9300
C3—H3	0.9300	C5—H5	0.9300
C7—H7A	0.9700		
			
C2—O1—H1A	110 (2)	C5—C6—H6	119.1
C8—N1—C7	117.0 (2)	C1—C6—H6	119.1
C8—N1—H1B	110.3 (19)	C8—C9—C10	121.4 (3)
C7—N1—H1B	110.4 (19)	C8—C9—H9	119.3
O1—C2—C1	118.1 (2)	C10—C9—H9	119.3
O1—C2—C3	121.4 (2)	C8—C13—C12	120.2 (3)
C1—C2—C3	120.4 (2)	C8—C13—H13	119.9
C6—C1—C2	117.9 (3)	C12—C13—H13	119.9
C6—C1—C7	120.8 (2)	C12—C11—C10	120.4 (3)
C2—C1—C7	121.3 (2)	C12—C11—Cl1	120.1 (3)
C9—C8—C13	118.6 (3)	C10—C11—Cl1	119.5 (3)
C9—C8—N1	118.7 (2)	C9—C10—C11	119.2 (3)
C13—C8—N1	122.7 (3)	C9—C10—H10	120.4
C4—C3—C2	119.5 (3)	C11—C10—H10	120.4
C4—C3—H3	120.2	C5—C4—C3	121.1 (3)
C2—C3—H3	120.2	C5—C4—H4	119.4
N1—C7—C1	111.6 (2)	C3—C4—H4	119.4
N1—C7—H7A	109.3	C11—C12—C13	120.2 (3)
C1—C7—H7A	109.3	C11—C12—H12	119.9
N1—C7—H7B	109.3	C13—C12—H12	119.9
C1—C7—H7B	109.3	C4—C5—C6	119.1 (3)
H7A—C7—H7B	108.0	C4—C5—H5	120.4
C5—C6—C1	121.8 (3)	C6—C5—H5	120.4
			
O1—C2—C1—C6	178.7 (2)	C13—C8—C9—C10	−0.1 (4)
C3—C2—C1—C6	1.9 (4)	N1—C8—C9—C10	177.9 (2)
O1—C2—C1—C7	−0.2 (4)	C9—C8—C13—C12	0.0 (4)
C3—C2—C1—C7	−177.0 (2)	N1—C8—C13—C12	−177.9 (2)
C7—N1—C8—C9	164.5 (2)	C8—C9—C10—C11	0.6 (5)
C7—N1—C8—C13	−17.7 (4)	C12—C11—C10—C9	−1.0 (5)
O1—C2—C3—C4	−179.2 (2)	Cl1—C11—C10—C9	179.0 (2)
C1—C2—C3—C4	−2.5 (4)	C2—C3—C4—C5	1.1 (4)
C8—N1—C7—C1	−174.1 (2)	C10—C11—C12—C13	0.8 (5)
C6—C1—C7—N1	83.6 (3)	Cl1—C11—C12—C13	−179.1 (2)
C2—C1—C7—N1	−97.5 (3)	C8—C13—C12—C11	−0.3 (5)
C2—C1—C6—C5	−0.1 (4)	C3—C4—C5—C6	0.7 (5)
C7—C1—C6—C5	178.8 (3)	C1—C6—C5—C4	−1.2 (5)

### Hydrogen-bond geometry (Å, °)

**Table:**

*D*—H···*A*	*D*—H	H···*A*	*D*···*A*	*D*—H···*A*
N1—H1B···O1^i^	0.86 (1)	2.20 (1)	3.038 (3)	165 (3)
O1—H1A···N1^ii^	0.85 (1)	1.93 (1)	2.777 (3)	171 (3)

Symmetry codes: (i) *x*−1, *y*, *z*; (ii) −*x*+2, −*y*+1, −*z*+1.
